# Phenome-wide association study identifies marked increased in burden of comorbidities in African Americans with systemic lupus erythematosus

**DOI:** 10.1186/s13075-018-1561-8

**Published:** 2018-04-10

**Authors:** April Barnado, Robert J. Carroll, Carolyn Casey, Lee Wheless, Joshua C. Denny, Leslie J. Crofford

**Affiliations:** 1 Department of Medicine, Vanderbilt University Medical Center, 1161 21st Avenue South, T3113 MCN, Nashville, TN 37232 USA; 2 Department of Biomedical Informatics, Vanderbilt University Medical Center, Nashville, TN USA; 3 Department of Medicine, Lehigh Valley Health Network, Allentown, PA USA; 4 Department of Dermatology, Vanderbilt University Medical Center, Nashville, TN USA

**Keywords:** Systemic lupus erythematosus, Electronic health records, Phenome-wide association study, Health disparities, Racial disparities

## Abstract

**Background:**

African Americans with systemic lupus erythematosus (SLE) have increased renal disease compared to Caucasians, but differences in other comorbidities have not been well-described. We used an electronic health record (EHR) technique to test for differences in comorbidities in African Americans compared to Caucasians with SLE.

**Methods:**

We used a de-identified EHR with 2.8 million subjects to identify SLE cases using a validated algorithm. We performed phenome-wide association studies (PheWAS) comparing African American to Caucasian SLE cases and African American SLE cases to matched non-SLE controls. Controls were age, sex, and race matched to SLE cases. For multiple testing, a false discovery rate (FDR) *p* value of 0.05 was used.

**Results:**

We identified 270 African Americans and 715 Caucasians with SLE and 1425 matched African American controls. Compared to Caucasians with SLE adjusting for age and sex, African Americans with SLE had more comorbidities in every organ system. The most striking included hypertension odds ratio (OR) = 4.25, FDR *p* = 5.49 × 10^− 15^; renal dialysis OR = 10.90, FDR *p* = 8.75 × 10^− 14^; and pneumonia OR = 3.57, FDR *p* = 2.32 × 10^− 8^. Compared to the African American matched controls without SLE, African Americans with SLE were more likely to have comorbidities in every organ system. The most significant codes were renal and cardiac, and included renal failure (OR = 9.55, FDR *p* = 2.26 × 10^− 40^) and hypertensive heart and renal disease (OR = 8.08, FDR *p* = 1.78 × 10^− 22^). Adjusting for race, age, and sex in a model including both African American and Caucasian SLE cases and controls, SLE was independently associated with renal, cardiovascular, and infectious diseases (all *p* < 0.01).

**Conclusions:**

African Americans with SLE have an increased comorbidity burden compared to Caucasians with SLE and matched controls. This increase in comorbidities in African Americans with SLE highlights the need to monitor for cardiovascular and infectious complications.

**Electronic supplementary material:**

The online version of this article (10.1186/s13075-018-1561-8) contains supplementary material, which is available to authorized users.

## Background

Health disparities are defined as “differences in the incidence, prevalence, mortality and burden of diseases and other adverse health conditions that exist among specific population groups in the US” [1]. Among rheumatic diseases, systemic lupus erythematosus (SLE) has one of the highest mortality rates and highest rates of health disparity [2]. SLE disproportionately affects African Americans, particularly female African Americans, who have nearly threefold higher incidence of SLE compared to Caucasians [3]. Female African Americans also have a younger age of onset and increased rates of renal disease compared to Caucasians [3, 4]. Female African Americans in the USA have the highest SLE mortality rates [5, 6]. Studies, however, have not fully examined differences in comorbidities in African Americans compared to Caucasians with SLE.

Racial disparities in SLE have mainly been studied using cohort and administrative database studies. Cohort studies typically have focused on SLE-related disease measures such as disease activity and may not capture other important comorbidities. Alternatively, administrative studies may not capture detailed data on a patient’s SLE disease course or comorbidities. Therefore, studies have not fully examined the impact of both the SLE disease course and comorbidities on outcomes. Electronic health records (EHRs) serve as an efficient and cost-effective discovery tool [7–9] to provide detailed data on both a patient’s SLE disease course and comorbidities. One method to harness the power of the longitudinal, clinical data in the EHR is the phenome-wide association study (PheWAS). Similar to the way a genome-wide association study (GWAS) scans across the genome, a PheWAS scans across diseases in the EHR, using aggregations of billing codes. PheWAS have uncovered novel genetic associations in multiple autoimmune diseases [10–13] and have found novel phenotypes with autoantibodies in rheumatoid arthritis [14–16]. PheWAS has also been validated across multiple EHRs and using orthogonal methods [11–13, 17, 18]. To the best of our knowledge, PheWAS have not been used in SLE to examine differences in comorbidities between African Americans and Caucasians with SLE. We hypothesized that PheWAS could take advantage of the longitudinal data in the EHR to systematically test for differences in comorbidities that would inform racial disparities in SLE.

## Methods

### Study population

After approval from the Institutional Review Board of Vanderbilt University Medical Center (VUMC), we identified potential SLE subjects in Vanderbilt’s Synthetic Derivative [19]. VUMC is a regional, tertiary care medical center. The Synthetic Derivative is a de-identified version of the EHR with over 2.8 million subjects with longitudinal data over several decades. The Synthetic Derivative contains all available information in the EHR such as diagnostic and procedure codes, demographics, inpatient and outpatient notes (including both subspecialty and primary care), laboratory values, radiology and pathology results, and medication orders. Outside records are not available in the Synthetic Derivative. The Synthetic Derivative is composed of approximately equal numbers of male and female individuals who are predominantly Caucasian (81%), reflecting the patient population of VUMC.

To identify SLE patients within the Synthetic Derivative, we used our validated EHR algorithm [20] of ≥ 4 counts of the SLE ICD-9 code (710.0) and a positive anti-nuclear antibody (ANA) with a titer ≥1:160 while excluding ICD-9 codes for systemic sclerosis (710.1) and dermatomyositis (710.3). This previously described algorithm [20] has a positive predictive value (PPV) of 89% and sensitivity of 86%.

Non-SLE controls were defined as subjects within the Synthetic Derivative who did not have ICD-9 codes for the 710.* heading “Diffuse diseases of connective tissue,” 714.* heading of “Rheumatoid Arthritis and other inflammatory polyarthropathies,” or ICD-10 codes under M05.* (“Rheumatoid Arthritis with rheumatoid factor”), M06.* (“Other rheumatoid arthritis”), M32.* (“SLE”), M33.* (“Dermatopolymyositis”), M34.* (“Systemic sclerosis”), M35.* (“Other systemic involvement of connective tissue”), and M36.* (“Systemic disorders of connective tissue in diseases classified elsewhere”). Controls were age (± 5 years), race, and sex matched in a 5:1 ratio to SLE cases to maximize power while allowing for close matching. Controls were “medical home” patients [21] who received longitudinal care at VUMC, defined as three outpatient visits within 5 years, to ensure similar density of records to the SLE cases. We examined age at time of analysis, age at first use of the SLE ICD-9 code, sex, and race. Race was derived from the EHR, which is a mixture of self-report and administrative entry. Prior studies have validated that these EHR race assignments reflect self-report and genetic ancestry [22]. Due to the small numbers of subjects with SLE who were Asian (*n* = 25) or Hispanic (n = 30), analyses were restricted to Caucasian and African American subjects with SLE, as PheWAS requires models to have at least 20 subjects for a particular code to be used in the model.

### Phenome-wide association studies and statistics

In PheWAS, the 18,000 ICD-9 codes are collapsed into 1800 PheWAS codes that represent distinct clinical diagnoses. The ICD-9 codes that are mapped to PheWAS codes are version 1.2 and available at http://phewascatalog.org. To be a case, we required the subject to have at least two instances of the PheWAS code on different days, at least 1 day apart. A subject is a control if there are no instances of the ICD-9 code for the given disease or related diseases. Subjects having only one instance of the code are excluded to eliminate the possibility of coding errors or preliminary diagnoses that may be ultimately ruled out [23]. For each PheWAS code, an unconditional logistic regression model was created with the option to add covariates with odds ratios (ORs) and 95% confidence intervals (CIs) reported. There must be at least 20 cases [10, 11] for the code to be used in the model. Analyses were performed and graphed in the PheWAS package [23] in R version 3.2.5. We performed (1) PheWAS comparing African Americans to Caucasians with SLE, adjusting for age and sex, (2) PheWAS comparing African American SLE cases to non-SLE, matched controls, and (3) PheWAS comparing Caucasian SLE cases to non-SLE, matched controls. We adjusted for multiple hypotheses testing using a false discovery rate (FDR) of 0.05. There were 478 testable phenotypes for the African American vs. Caucasian SLE PheWAS, 430 for the African American SLE cases vs. matched controls PheWAS, and 732 for the Caucasian SLE cases vs. matched controls. For the most significant codes, we performed conditional logistic regression using SLE cases and matched controls (including both African Americans and Caucasians) to calculate an OR and 95% CI for the association between the codes and SLE, adjusting for age, sex, and race. We assessed for differences in demographics in African Americans vs. Caucasians with SLE using the Mann-Whitney U test for continuous variables, as there were non-normal distributions in the data, and the chi-square or Fisher’s exact test for categorical variables. Two-sided *p* values <0.05 were considered to indicate statistical significance. Analyses were conducted using IBM SPSS software, version 24.0 (SPSS).

## Results

### Study population

Using our validated algorithm [20], we identified 1097 subjects with SLE including 3 with missing race/ethnicity data, 2 as “other” and 52 as “unknown” race/ethnicity, 25 Asians, 30 Hispanics, 270 African Americans, and 715 Caucasians. We then restricted analyses to the African American and Caucasian subjects with SLE. Both African American and Caucasian subjects with SLE were predominantly female (89% vs. 90%, *p* = 0.83). Compared to Caucasians, African Americans were younger (44 ± 17 vs. 53 ± 17, *p* < 0.001) and had an earlier age at first SLE code (35 ± 16 vs. 43 ± 17, *p* < 0.001) with similar mean years of EHR follow up (9 ± 5 vs. 10 ± 5, *p* = 0.10) (Table 1).

**Table 1:** Demographics of African Americans and Caucasians with SLE

Demographics	African Americans (*n* = 270)	Caucasians (*n* = 715)	*p* value^a^
Current age, mean ± SD	44 ± 17	53 ± 17	<0.001
Age at first SLE code (710.0), mean ± SD	35 ± 16	43 ± 17	<0.001
Female (%)	89	90	0.83
Years of follow up in the EHR, mean ± SD	9 ± 5	10 ± 5	0.10

*SLE* systemic lupus erythematosus, *EHR* electronic health record

^a^Mann-Whitney U or Fisher’s exact test

### PheWAS of Caucasians and African Americans with SLE

**Fig. 1:**
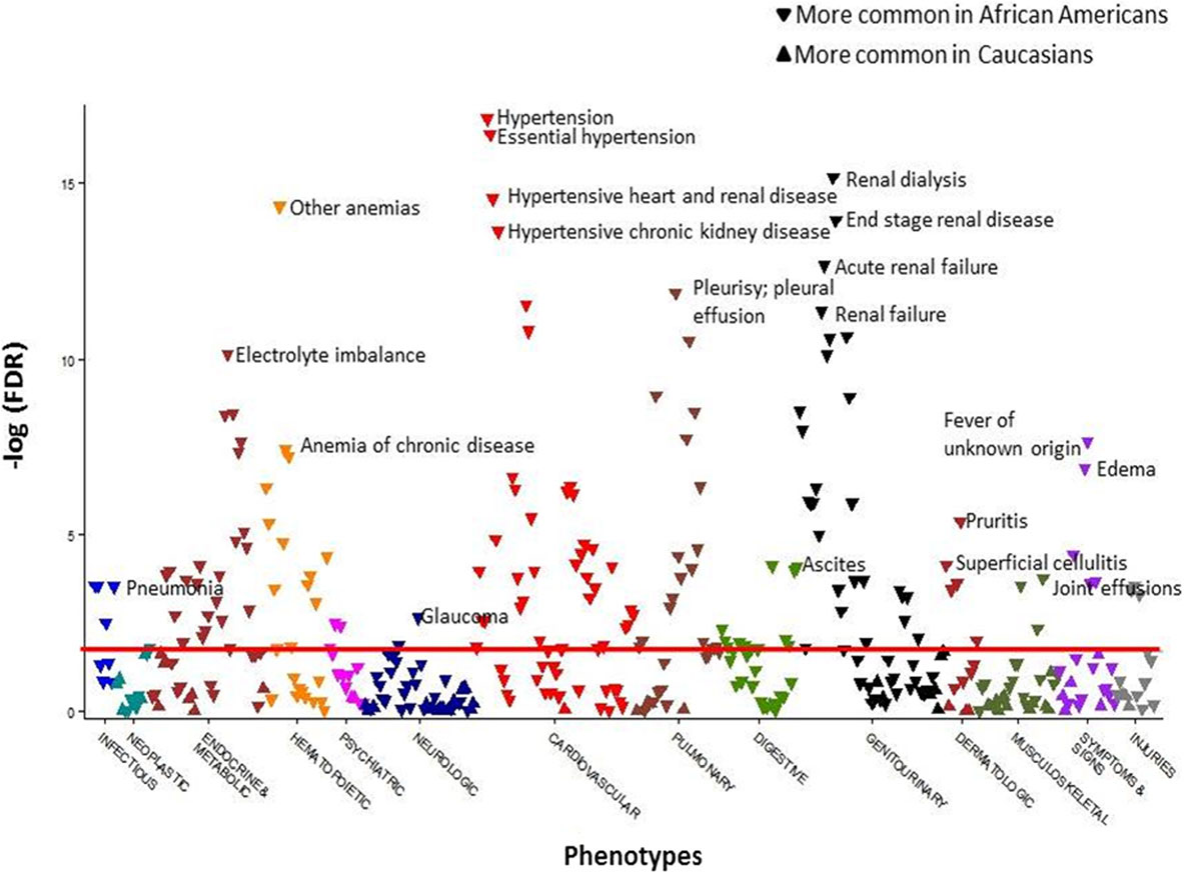
Increased comorbidities across all organ systems in African Americans with systemic lupus erythematosus (SLE) compared to Caucasians using phenome-wide association studies (PheWAS). The x axis represents the PheWAS codes that are mapped to ICD-9 codes, organized and color-coded by organ system. The y axis represents the level of significance. Each triangle represents a PheWAS code. African Americans are the reference group. Triangles pointing down represent codes more common in African Americans. Triangles pointing up represent codes more common in Caucasians. The PheWAS was adjusted for age and sex, and the horizontal red line represents the false discovery rate (FDR) of 0.05. There were 163 codes that met the FDR of 0.05. African Americans with SLE had more codes compared to Caucasians with SLE for comorbidities across all organ systems. The most significant codes for each organ system are labeled

In the PheWAS comparing Caucasians to African Americans with SLE, adjusting for current age and sex, there were 163 codes that met the FDR of 5% (Additional file 1: Table S1). Compared to Caucasians, African Americans had codes for more comorbidities in every organ system (Fig. 1). The most significant codes, seen more frequently in the African Americans with SLE, were cardiac and renal. The most significant cardiac code was hypertension (OR = 4.25, 95% CI 3.05–5.92, FDR *p* = 5.49 × 10^− 15^). Other significant cardiac codes covered a range of cardiovascular comorbidities including congestive heart failure (CHF) (FDR *p* = 5.63 × 10^− 6^), cerebrovascular disease (CVD) (FDR *p* = 4.23 × 10^− 4^), thromboembolism (FDR *p* = 4.47 × 10^− 4^), peripheral vascular disease (PVD) (FDR *p* = 0.04), coronary artery disease (CAD) (FDR *p* = 0.04), and arrhythmias (FDR *p* = 0.04) (Table 2). The most significant renal code was renal dialysis (OR = 10.90, 6.11–19.48, FDR *p* = 8.75 × 10^− 14^) (Table 2). Other codes included acute renal failure (FDR *p* = 1.05 × 10^− 11^), chronic renal failure (FDR *p* = 7.08 × 10^− 10^), renal transplant (FDR *p* = 2.32 × 10^− 8^), and nephritis related codes. In addition to cardiovascular and renal disease, African Americans with SLE were also more likely to have codes related to infection. The most significant code was pneumonia (OR = 3.57, 2.57–5.38, FDR *p* = 2.32 × 10^− 8^) with other significant codes related to cellulitis, pyelonephritis, and bacteremia (Table 2).

**Table 2:** Selected cardiac, renal, and infectious codes from the PheWAS of African Americans compared to Caucasians with SLE

PheWAS code name (code number)	Phenotype present (≥ 2 instances of PheWAS code)^a^	Phenotype absent (0 instances of PheWAS code)^a^	Adjusted odds ratio for current age and sex (95% CI)	False discovery rate *p*^b^
Selected cardiac codes				
Hypertension (401)	423	494	African American, 4.25 (3.05–5.92) Caucasian, 1.00 (ref)	5.49 × 10^− 15^
Other forms of chronic heart disease (414)	30	790	9.10 (3.94–20.98)	2.69 × 10^− 6^
Congestive heart failure (428)	86	702	3.53 (2.15–5.78)	5.63 × 10^−6^
Cerebrovascular disease (433)	85	841	2.80 (1.69–4.65)	4.23 × 10^−4^
Other venous embolism and thrombosis (452)	89	741	2.58 (1.62–4.13)	4.47 × 10^−4^
Heart valve disorders (395)	68	794	2.89 (1.69–4.95)	5.52 × 10^−4^
Pulmonary embolism and infarction, acute (415.11)	28	785	3.73 (1.69–8.23)	4.03 × 10^− 3^
Cardiomyopathy (425)	27	823	2.87 (1.29–6.41)	0.03
Peripheral vascular disease (443.9)	25	738	3.16 (1.27–7.90)	0.04
Atherosclerosis (440)	24	738	2.99 (1.22–7.37)	0.04
Cardiac dysrhythmias (427)	171	684	1.60 (1.08–2.36)	0.04
Selected renal codes				
Renal dialysis (585.31)	73	600	10.90 (6.11–19.48)	8.75 × 10^−14^
End stage renal disease (585.32)	76	600	8.57 (4.97–14.79)	7.15 × 10^−13^
Hypertensive chronic kidney disease (401.22)	103	494	6.63 (4.08–10.77)	1.24 × 10^−12^
Acute renal failure (585.1)	149	600	4.43 (2.98–6.60)	1.05 × 10^−11^
Chronic renal failure (CKD) (512)	169	600	3.64 (2.49–5.33)	7.08 × 10^−10^
Kidney replaced by transplant (587)	49	600	7.67 (3.98–14.78)	2.32 × 10^− 8^
Nephritis; nephrosis; renal sclerosis (512.7)	188	600	3.03 (2.10–4.37)	5.60 × 10^− 8^
Glomerulonephritis (580.1)	90	600	4.10 (2.53–6.65)	1.66 × 10^−7^
Selected infection codes				
Pneumonia (480)	128	701	3.57 (2.37–5.38)	2.32 × 10^−8^
Superficial cellulitis and abscess (681)	92	793	2.56 (1.61–4.06)	4.30 × 10^−4^
Pyelonephritis (590)	29	538	4.45 (2.03–9.74)	9.20 × 10^− 4^
Bacteremia (038.3)	58	894	2.83 (1.62–4.95)	1.18 × 10^−3^
Candidiasis (112)	72	806	2.57 (1.54–4.29)	1.23 × 10^− 3^
Cellulitis and abscess of trunk (681.7)	22	793	5.62 (2.18–14.45)	1.41 × 10^−3^
Sepsis and SIRS (994)	66	888	1.88 (1.10–3.20)	0.05

*SLE* systemic lupus erythematosus, *PheWas* phenome-wide association studies, *SIRS* systemic inflammatory response syndrome

^a^Phenotype present indicates subjects who had the code listed on at least two instances vs. phenotype absent indicates subjects who did not have the code or related codes. Subjects with one instance of a code are excluded, so the total number of subjects for each PheWAS code does not add up to the 1097 subjects with SLE

^b^Codes listed met the false discovery rate of 0.05

Compared to Caucasians with SLE, African Americans with SLE were more likely to have codes related to SLE criteria (Additional file 1: Figure S1 and Table S2). The most significant codes were pleurisy/pleural effusion (OR = 4.39, 2.92–6.62, FDR *p* = 5.53 × 10^− 11^) and nephritis; nephrosis; renal sclerosis (OR = 3.03, 2.10–4.37, FDR *p* = 5.60 × 10^− 8^). African Americans had more codes related to serositis including pericarditis (FDR *p* = 5.52 × 10^− 4^) and hematologic abnormalities such as thrombocytopenia (FDR *p* = 7.03 × 10^− 4^). African Americans were also more likely to have codes related to arthritis including joint effusions (FDR *p* = 8.41 × 10^− 4^) and neuropsychiatric involvement with encephalopathy (FDR *p* = 0.04).

### PheWAS of African Americans with SLE and African American controls

To examine the increased cardiac, renal, and infectious comorbidities seen in the African Americans with SLE, we compared African Americans with SLE to African Americans without SLE as our controls, given known health disparities in African Americans. We identified 1425 control subjects who were age (± 5 years), sex, and race matched in a 5:1 ratio to the 270 African American SLE case subjects. African American SLE case subjects and their matched controls had similar mean current age (44 ± 17 vs. 44 ± 16, *p* = 0.97) and were predominantly female (89% vs. 93%, *p* = 0.11).

**Fig. 2:**
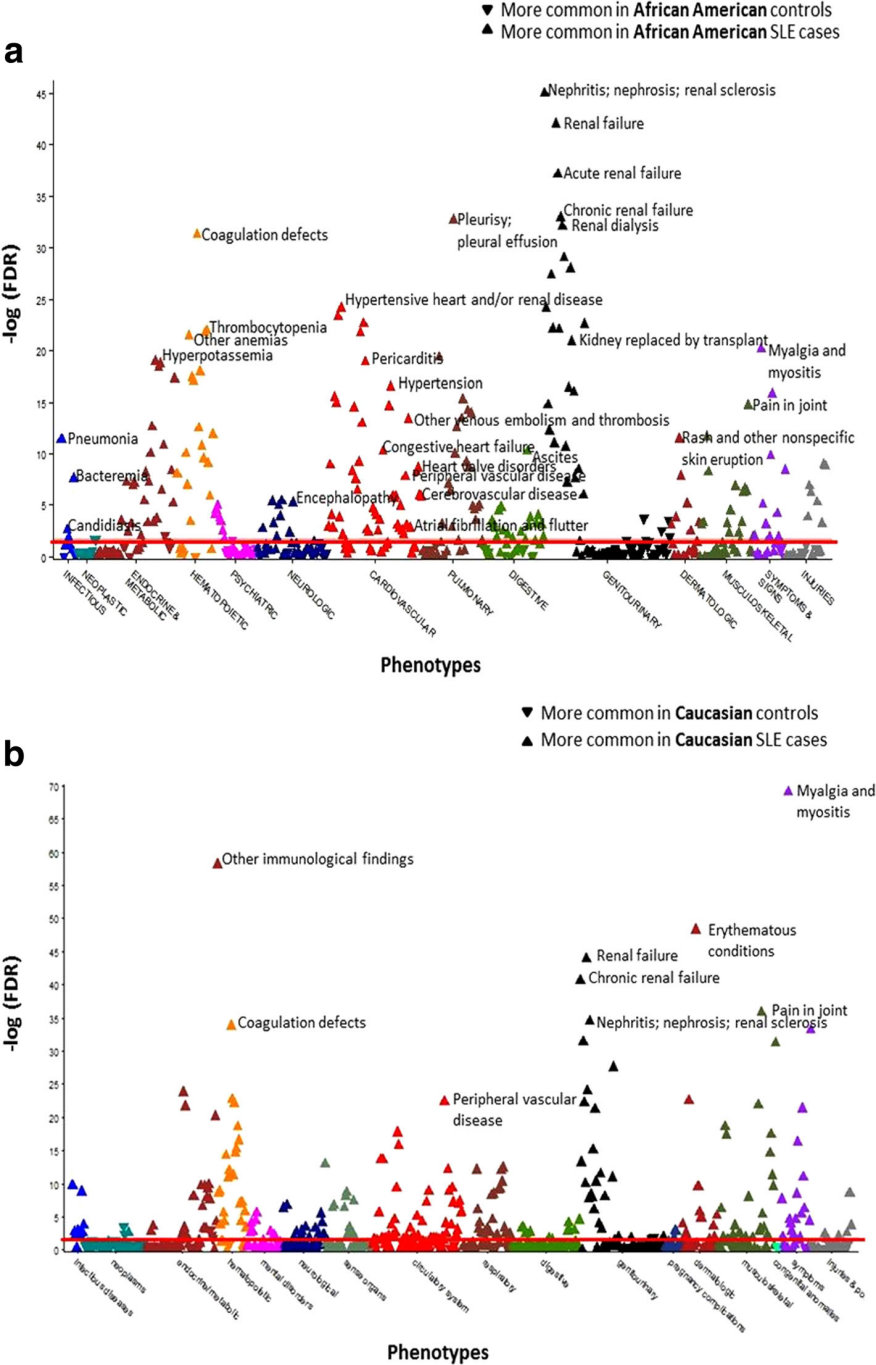
Increased comorbidities in case subjects with systemic lupus erythematosus (SLE) compared to matched controls using phenome-wide association studies (PheWAS). The x axis represents the PheWAS codes that are mapped to ICD-9 codes, organized and color-coded by organ system. The y axis represents the level of significance. Each triangle represents a PheWAS code. Controls are the reference group and were age (± 5 years), sex, and race matched. Triangles pointing up represent codes more common in subjects with SLE. Triangles pointing down represent codes more common in non-SLE matched controls. The horizontal red line represents the false discovery rate (FDR) of 0.05. **a** PheWAS comparing African American SLE cases to African American controls. African Americans with SLE had more codes compared to matched controls for comorbidities across all organ systems, particularly cardiac and renal. The most significant codes for each organ system are labeled. **b** PheWAS comparing Caucasian SLE cases to Caucasian controls. Caucasians with SLE had more renal and SLE-related codes compared to controls. The most significant codes for organ systems are labeled

Using PheWAS to compare African Americans with SLE to matched controls, there were 213 codes that met the FDR of 5%. Compared to controls, African American SLE case subjects had more codes related to comorbidities in every organ system (Fig. 2a). African American SLE case subjects were more likely to have codes related to cardiovascular, renal, and infectious diseases. The most significant renal code was renal failure (OR = 9.55, 6.91–13.18, FDR *p* = 2.26 × 10^− 40^) with other significant codes including renal dialysis, end-stage renal disease, and renal transplant (Additional file 1: Table S3). The most significant cardiac code included hypertensive heart and/or renal disease (OR = 8.08, 5.39–12.11, FDR *p* = 1.78 × 10^− 22^). Other significant codes covered a range of cardiovascular disease comorbidities including thromboembolism (FDR *p* = 4.74 × 10^− 13^), CHF (FDR *p* = 4.17 × 10^− 10^), PVD (FDR *p* = 8.84 × 10^− 8^), atrial fibrillation (AF) (FDR *p* = 1.91 × 10^− 4^), and CAD (FDR *p* = 0.02). The most significant infection code was pneumonia OR = 5.77 (3.97–8.39, FDR *p* = 8.66 × 10^− 19^). Other significant codes included sepsis (FDR *p* = 7.49 × 10^− 9^), candidiasis (FDR *p* = 1.34 × 10^− 7^), and cellulitis (FDR *p* = 5.41 × 10^− 4^).

As SLE cases had significantly longer EHR follow up compared to controls, we conducted a sensitivity analysis adjusting for years of EHR follow up to see if the longer follow up could account for the higher risk of comorbidities in the SLE cases. Adjusting for years of follow up, case subjects with SLE were still more likely to have codes related to cardiovascular, renal, and infectious diseases. The most significant renal, cardiovascular, and infectious diseases were relatively unchanged with renal failure (OR = 10.12, 95% CI 7.23–14.17, FDR *p* = 7.33 × 10^− 39^), hypertensive heart and/or renal disease (OR = 8.51, 5.59–12.95, FDR *p* = 6.90 × 10^− 22^), and pneumonia (OR = 6.09, 4.13–9.00, FDR *p* = 2.11 × 10^− 19^).

Compared to matched controls, African American subjects with SLE had more codes related to American College of Rheumatology (ACR) SLE criteria [24] with the most significant codes related to renal criteria including nephritis; nephrosis; renal sclerosis (OR = 50.53, 29.40–86.86, FDR *p* = 4.77 × 10^− 43^) and glomerulonephritis (OR = 72.36, 32.04–163.39, FDR *p* = 2.88 × 10^− 23^). Other significant codes represented serositis, arthritis, and hematologic and neuropsychiatric involvement (Additional file 1: Table S4).

### PheWAS of Caucasians with SLE and Caucasian controls

We compared 715 Caucasian subjects with SLE to 3731 controls who were age (± 5 years), sex, and race matched in a 5:1 ratio. Caucasian subjects with SLE and their matched controls had similar mean current age (53 ± 16 vs. 53 ± 16, *p* = 0.86) and were predominantly female (90% vs. 90%, *p* = 0.95). Using PheWAS to compare Caucasians with SLE to matched Caucasian controls, there were 262 codes that met the FDR of 5%. Compared to the controls, the Caucasians with SLE had more codes that represented SLE-related and renal comorbidities (Fig. 2b, Additional file 1: Table S5). The most significant code was myalgia and myositis unspecified (OR = 8.30, 95% CI 6.57–10.49, FDR *p* = 2.90 × 10^− 67^), which signifies fibromyalgia in our EHR, based on a prior study [15]. Other significant codes included a dermatologic group of codes called erythematous conditions (OR = 14.01, 9.91–19.81, FDR *p* = 5.01 × 10^− 48^), which includes a code for discoid lupus, and renal failure (OR = 6.16, 4.80–7.91, FDR *p* = 7.91 × 10^− 44^). Other renal codes included nephritis; nephrosis; renal sclerosis (FDR *p* = 4.88 × 10^− 28^) and chronic kidney disease (FDR *p* = 3.17 × 10^− 21^). Notably, there were fewer cardiac and infection codes compared to the PheWAS of African Americans with SLE and African American controls. The most significant cardiac code was peripheral vascular disease (OR = 8.19, 5.40–12.42, FDR *p* = 1.55 × 10^− 21^). The most significant infection code was pneumonia (OR = 3.26, 2.37–12.42, FDR *p* = 6.32 × 10^− 12^). As expected, similar to the PheWAS of the African Americans with SLE and African American controls, codes that represent ACR SLE criteria [24] were more common in the Caucasians with SLE compared to the Caucasian controls. Significant codes included pain in joint, nephritis/nephrosis/renal sclerosis, thrombocytopenia, and pericarditis (Additional file 1: Table S5). In a sensitivity analysis, results remain unchanged when adjusted for years of EHR follow up.

### Conditional logistic regression models

**Fig. 3:**
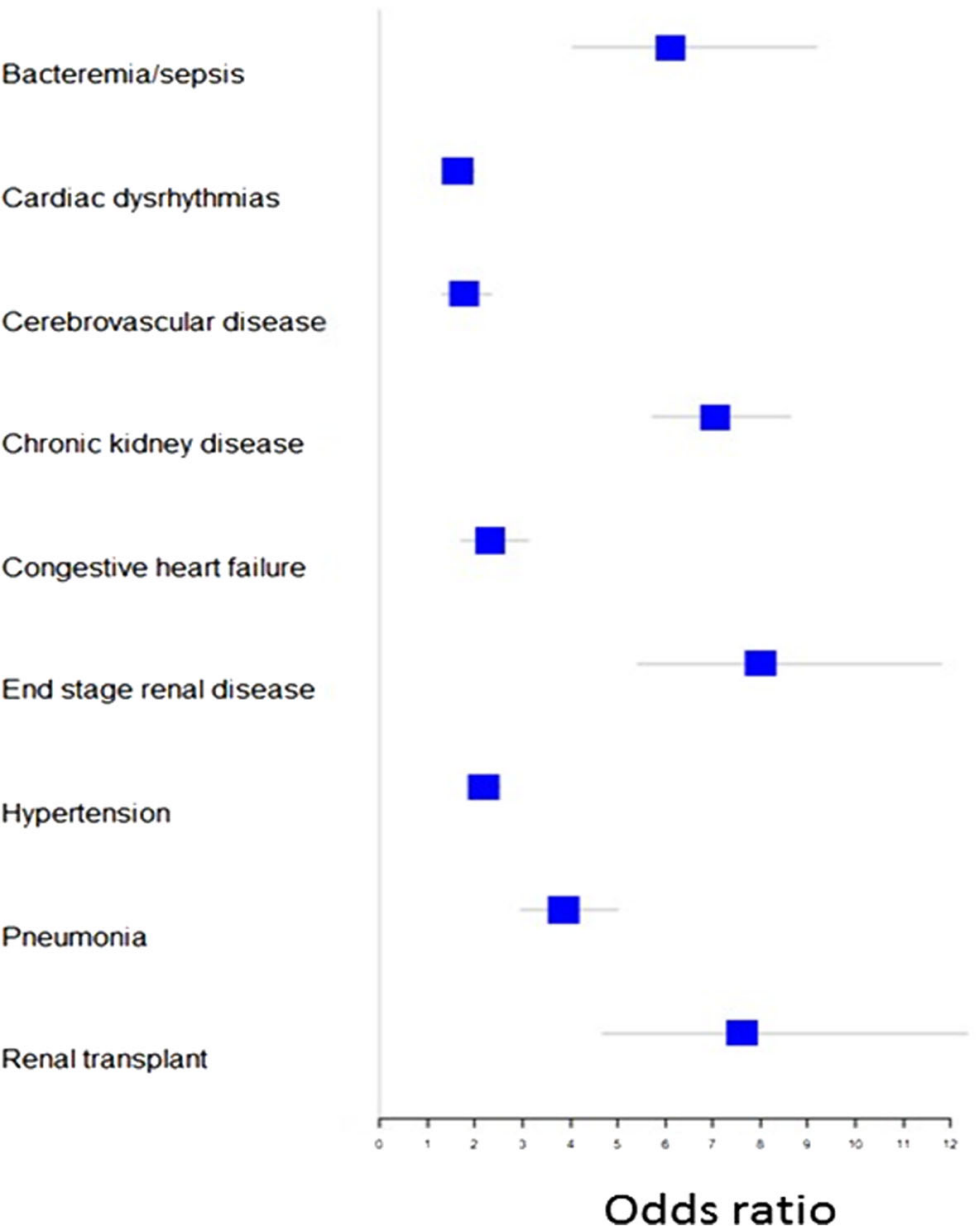
Conditional logistic regression models of systemic lupus erythematosus (SLE) case subjects and matched controls. Conditional logistic regression models were created with SLE case subjects and matched controls (including both African Americans and Caucasians) to examine the association between SLE and phenome-wide association study codes, adjusting for age, race, and sex. Odds ratios are shown with horizontal lines depicting 95% confidence intervals

Using a conditional logistic regression model of SLE cases and matched controls (including both Caucasians and African Americans), we examined the association of SLE with PheWAS codes adjusting for age, race, and sex. SLE was significantly associated with hypertension (OR = 2.18, 95% 1.86–2.55, *p* < 0.01), CHF (OR = 2.33, 1.73–3.13, *p* = 2.2 × 10^− 8^), CVD (OR = 1.76, 1.34–2.32, *p* = 5.8 × 10^− 5^), and cardiac dysrhythmias (OR = 1.63, 1.34–1.97, *p* = 9.0 × 10^− 7^) (Fig. 3, Additional file 1: Table S6). SLE was also independently associated with the following renal codes: chronic kidney disease (OR = 7.04, 5.73–8.65, *p* < 0.001), end-stage renal disease (OR = 8.00, 5.43–11.80, *p* < 0.001), and renal transplant (OR = 7.61, 7.26–7.99, *p* = 1.1 × 10^− 16^). Lastly, SLE disease status was independently associated with infections including pneumonia (OR = 3.87, 2.98–5.01, *p* < 0.001) and sepsis/bacteremia (OR = 6.10, 4.05–9.19, *p* < 0.001).

## Discussion

Using PheWAS in a large cohort of 1097 subjects with SLE using EHR data with decades of follow up, we uncovered an increased burden of comorbidities across all organ systems among African Americans compared to Caucasians with SLE. African Americans with SLE were two to four times more likely to have renal disease, cardiovascular disease, and infections. To the best of our knowledge, this is the first study to use PheWAS to examine racial disparities between African Americans and Caucasians with SLE. Since some comorbidities are more frequent in non-SLE African Americans compared with Caucasians [25], we determined the impact of SLE on comorbidities in African Americans. Compared to matched African American controls, African American subjects with SLE were significantly more likely to have comorbidities in all organ systems, notably in renal, cardiovascular, and infectious diseases.

PheWAS enables a systematic assessment of diverse phenotypes in the EHR, building upon both traditional cohort and administrative database studies. PheWAS has the potential to capture both SLE disease-related data such as ACR SLE criteria [24], as well as other comorbidities. Data on these comorbidities may not be collected in traditional cohort studies, while administrative database studies may not adequately capture SLE-related data. Further, administrative databases can have a fairly short duration of follow up [26, 27]. In contrast, our EHR has follow up over several decades with subjects with SLE having on average 9 years of follow up [20]. PheWAS has the power to capture diverse comorbidities in the EHR and uncover how these comorbidities contribute to racial disparities in SLE.

Compared to Caucasians, African American patients with SLE have increased renal disease. These disparities have been attributed to both genetic and non-genetic factors such as the environment and socioeconomic status [28]. As expected, we observed an increased burden of renal disease in African Americans compared to Caucasians with SLE, which agrees with findings in prior SLE cohorts [29–34]. While PheWAS confirmed known renal disparities in African Americans with SLE, it also uncovered an increased cardiovascular disease burden, which has not been previously well-described. African Americans with SLE were three times more likely to have CAD compared to Caucasians with SLE. Administrative studies have shown increased CAD in African Americans compared to Caucasians with SLE when restricting analyses to inpatient encounters and subsets of patients with SLE [35, 36]. Our study builds upon these studies by including all patients with SLE and capturing CAD in both inpatient and outpatient encounters. In contrast to these administrative database studies, two cohort studies did not find increased CAD in African Americans compared to Caucasians with SLE [37–39]. These differences could be due to different SLE patient populations. In contrast to traditional SLE cohorts, our EHR SLE cohort may represent a more community-based group of patients with SLE. Further, unless a cohort study collects data on a specific outcome, these outcomes may be underreported, as they may rely on either patient report or traditional methods that often focus on disease activity measures. These SLE cohorts also had a low frequency of CAD, myocardial infarction, and CVD events, with one study having only 34 patients with any vascular event [39]. These low-frequency events may have made these studies underpowered to detect differences in CAD in African Americans compared to Caucasians with SLE. In our EHR cohort, looking across multiple codes that captured CAD, we had 177 events.

In addition to CAD, African Americans with SLE were three times more likely to have CHF and CVD and more than four times more likely to have hypertension compared to Caucasians. There are fewer studies comparing risk of these cardiovascular diseases in African Americans to Caucasians with SLE, with mixed results [36, 37]. Specifically, in one cohort, there were no differences in rates of CVD and PVD comparing African Americans to Caucasians [39]. This study included only 18 subjects with CVD and 5 with PVD in contrast to approximately 223 subjects with CVD and 25 with PVD in our study [39].

Beyond the increased renal and cardiac disease burden, African Americans with SLE had an increase in infectious diseases compared to Caucasians with SLE. African Americans were more than 3.5 times more likely to have pneumonia and twice as likely to have bacteremia and sepsis. Our study agrees with two studies using the Medicaid administrative database that identified an increased risk of serious infections in African Americans compared to Caucasians with SLE, with the most common being bacteremia, pneumonia, and cellulitis [26, 27]. Our study builds upon these studies by including both inpatient and outpatient infections and offering a longer follow up of 9 years compared to the mean follow up of the studies of 2.5 years.

To account for racial differences in comorbidities, we compared African Americans with SLE to matched African American controls, particularly since many of these comorbidities are more common in African Americans. As expected, African Americans with SLE had more codes related to ACR SLE criteria [24] showing that PheWAS can identify SLE disease characteristics in the EHR. Compared to matched controls, African Americans with SLE also had more comorbidities across all organ systems. Notably, African Americans with SLE were more likely to have codes for chronic kidney disease (CKD), end-stage renal disease (ESRD), and renal transplant. Using a conditional logistic regression model with SLE cases and matched controls (including both Caucasians and African Americans), adjusting for age, sex, and race, SLE remained independently associated with CKD, ESRD, and renal transplant suggesting that African American race was not the sole driver for increased renal disease.

Compared to matched controls, African Americans with SLE also had more codes for CAD, CVD, PVD, and arrhythmias. While a twofold to threefold increase in CAD has been described in subjects with SLE compared to population controls [40], there are few data comparing CAD events in African Americans with SLE compared to matched controls. One of the largest US population-based studies, the Nurses’ Health study, compared rates of CAD in participants with and without SLE showing a twofold to threefold increase in CAD events [41]. Notably, the cohort was all female and 95% Caucasian [41]. Our study is unique in that it included both male and female SLE patients and focused on African Americans. For other cardiac comorbidities, there are few studies comparing African American patients with SLE to matched controls [41, 42]. Further, studies often restrict analyses to subsets of patients with SLE [41, 42] while no studies directly compare patients with SLE to controls for PVD [43, 44] and AF. Our study builds upon large population-based studies by including male subjects and African Americans with SLE, who are often understudied and have adverse outcomes [45]. Our study also demonstrates novel findings of increased PVD and AF in African American patients with SLE compared to controls.

African Americans with SLE also had increased risk of multiple infections compared to matched controls. This increased risk of infection in SLE is likely due to immunosuppressant medications, the disease itself, or an interaction between these factors [46–48]. Two recent studies using the US Medicaid database investigated infection rates among different SLE patients but did not compare patients with SLE to matched controls [26, 27]. Our study establishes an increased risk of infection in African Americans with SLE compared to matched controls.

Our EHR-based PheWAS study has limitations. We used a previously validated algorithm to identify patients with SLE with a positive predictive value (PPV) of 89% and sensitivity of 86% [20]. Despite this algorithm’s strong test characteristics, we may have captured some subjects who do not have a SLE diagnosis. Our clinical EHR data, in contrast to prospective cohort studies, does not contain disease activity and damage measures such as the Systemic Lupus Erythematosus Disease Activity Index (SLEDAI) [49] and Systemic Lupus International Collaborating Clinics Damage Index (SDI) [50], as these measures are not collected routinely in clinical practice. Thus, we cannot adjust for disease activity or damage in PheWAS. EHR-based algorithms that assess treatment response in inflammatory bowel disease [51] and CAD risk in inflammatory bowel disease and RA [52] have been created. Currently, however, there are no published, EHR-based algorithms assessing disease severity and activity in autoimmune diseases. Future directions include developing these algorithms in SLE. Next, this PheWAS was performed using billing codes at Vanderbilt only. Patients can receive care in multiple healthcare systems, which may not be documented in Vanderbilt’s EHR. These potential missed diagnoses, however, would bias us to the null result. Missing data could be non-randomly distribute, with more occurring in the controls in whom EHR follow up was shorter compared to patients with SLE. We adjusted for EHR follow-up time, which did not alter our main findings. Last, our study was performed using a single institution’s EHR, potentially limiting generalizability of our results to other patients with SLE. Using an EHR-based cohort to study SLE, however, may capture a wider net of patients with SLE that are more representative of the community compared to patients with SLE recruited into a cohort. We did not have sufficient numbers of Hispanics or Asians, reflecting the demographics of middle Tennessee, to study patients with SLE with these ethnicities in our PheWAS. However, our EHR cohort included male subjects and African Americans with SLE, who are often understudied [45]. We acknowledge that the African American population in the USA is admixed, and these findings associated with the race construct could represent cultural and socioeconomic factors as well as genetic ancestry. Unfortunately, our de-identified resource does not contain socioeconomic data such as income level or insurance coverage, so we are unable to adjust for these factors in our PheWAS.

## Conclusion

Using PheWAS, we demonstrated an increased burden of comorbidities in African Americans with SLE compared to Caucasians with SLE and matched controls, including a spectrum of renal, cardiovascular, and infectious diseases. These findings suggest that clinicians managing patients with SLE should not only screen for SLE disease manifestations but also have suspicion of multiple cardiovascular and infectious diseases in their workup of common signs and symptoms to ensure appropriate and timely referrals and management. This high comorbidity burden in SLE, particularly in African Americans, argues for the need for access to care to not only rheumatology but also to primary and other subspecialty care. Further, this study demonstrates that an EHR-based approach can build upon traditional cohort and administrative database studies to examine racial disparities in SLE.

## Additional file

Additional file 1: **Table S1.** Significant codes from the PheWAS of African Americans vs. Caucasians with SLE. **Figure S1.** Selected SLE disease criteria codes in the PheWAS of African Americans and Cauasians with SLE. **Table S2.** Selected SLE criteria codes from the PheWAS of African Americans and Caucasians with SLE. **Table S3.** Selected codes related to renal, cardiovascular disease, and infection from the PheWAS of African American SLE cases compared to matched African American controls. **Table S4.** Selected codes related to SLE criteria from the PheWAS of African American SLE cases and matched African American controls. **Table S5.** Selected codes from the PheWAS of Caucasian SLE cases compared to matched Caucasian controls. **Table S6.** Conditional logistic regression models with SLE cases and matched controls. (DOCX 134 kb)

## Abbreviations

95% CI: 95% Confidence intervals
ACR: American College of Rheumatology
AF: Atrial fibrillation
ANA: Anti-nuclear antibody
CAD: Coronary artery disease
CHF: Congestive heart failure
CKD: Chronic kidney disease
CVD: Cerebrovascular disease
EHR: Electronic health record
ESRD: End-stage renal disease
FDR: False discovery rate
GWAS: Genome-wide association study
OR: Odds ratio
PheWAS: Phenome-wide association study
PPV: Positive predictive value
PVD: Peripheral vascular disease
SDI: Systemic Lupus International Collaborating Clinics Damage Index
SLE: Systemic lupus erythematosus
SLEDAI: Systemic Lupus Erythematosus Disease Activity Index
VUMC: Vanderbilt University Medical Center
