# Supplementary material for: Phenome-wide association study identifies marked increased in burden of comorbidities in African Americans with systemic lupus erythematosus
Source: Arthritis Res Ther. 2018 Apr 10;20:69. doi: 10.1186/s13075-018-1561-8 (PMC5894248; doi:10.1186/s13075-018-1561-8)
Supplement: Supplementary file 1 — Table S1. Significant codes from the PheWAS of African Americans vs. Caucasians with SLE. Figure S1. Selected SLE disease criteria codes in the PheWAS of African Americans and Cauasians with SLE. Table S2. Selected SLE criteria codes from the PheWAS of African Americans and Caucasians with SLE. Table S3. Selected codes related to renal, cardiovascular disease, and infection from the PheWAS of African American SLE cases compared to matched African American controls. Table S4. Selected codes related to SLE criteria from the PheWAS of African American SLE cases and matched African American controls. Table S5. Selected codes from the PheWAS of Caucasian SLE cases compared to matched Caucasian controls. Table S6. Conditional logistic regression models with SLE cases and matched controls. (DOCX 134 kb) [file 13075_2018_1561_MOESM1_ESM.docx]

**Table S1. Significant codes from the PheWAS of African Americans vs. Caucasians with SLE.**

| **PheWAS Code Name**  (Code Number) | **Phenotype Present**  (≥ 2 instances of code^)£^ | **Phenotype**  **Absent**  (0 instances of code)^£^ | **Adjusted Odds Ratio for current age and sex**  **(95% CI)** | **False Discovery Rate p*** |  |  |  |  |
| --- | --- | --- | --- | --- | --- | --- | --- | --- |
| Hypertension  (401) | 423 | 494 | **African American:** 4.25 (3.05 – 5.92)  **Caucasian**: 1.00 (ref) | 5.49 x 10^-15^ |  |  |  |  |
| Essential hypertension (401.1) | 408 | 494 | 4.24  (3.03 – 5.94) | 7.68 x 10^-15^ |  |  |  |  |
| Renal dialysis  (585.31) | 73 | 600 | 10.90  (6.11 -19.48) | 8.75 x 10^-14^ |  |  |  |  |
| Hypertensive heart and/or renal disease (401.2) | 119 | 494 | 6.41  (4.04 -10.16) | 2.66 x 10^-13^ |  |  |  |  |
| Other anemias  (285) | 266 | 557 | 3.86  (2.75 – 5.41) | 3.40 x 10^-13^ |  |  |  |  |
| End stage renal disease (585.32) | 76 | 600 | 8.57  (4.97 – 14.79) | 7.15 x 10^-13^ |  |  |  |  |
| Hypertensive chronic kidney disease  (401.22) | 103 | 494 | 6.63  (4.08 – 10.77) | 1.24 x 10^-12^ |  |  |  |  |
| Acute renal failure  (585.1) | 149 | 600 | 4.43  (2.98 – 6.60) | 1.05 x 10^-11^ |  |  |  |  |
| Pleurisy/pleural effusion (507) | 127 | 700 | 4.39  (2.92 – 6.62) | 5.53 x 10^-11^ |  |  |  |  |
| Cardiomegaly  (416) | 77 | 785 | 6.35  (3.78 – 10.66) | 1.12 x 10^-10^ |  |  |  |  |
| Renal failure  (585) | 252 | 600 | 3.25  (2.33 – 4.54) | 1.55 x 10^-10^ |  |  |  |  |
| Nonspecific chest pain (418) | 303 | 559 | 3.08  (2.22 – 4.27) | 5.03 x 10^-10^ |  |  |  |  |
| Other disorders of the kidney and ureters (418) | 106 | 600 | 4.66  (2.97 – 7.30) | 6.48 x 10^-10^ |  |  |  |  |
| Chronic renal failure (CKD) (585.3) | 169 | 600 | 3.64  (2.49 – 5.33) | 7.08 x10^-10^ |  |  |  |  |
| Other symptoms of respiratory system (512) | 357 | 490 | 3.06  (2.20 – 4.25) | 7.61 x 10^-10^ |  |  |  |  |
| Electrolyte imbalance (276.1) | 160 | 651 | 3.56  (2.43 – 5.22 | 1.64 x 10^-9^ |  |  |  |  |
| Renal failure NOS (585.2) | 50 | 600 | 9.03  (4.66 – 17.51) | 1.64 x 10^-9^ |  |  |  |  |
| Pneumonia (480) | 128 | 701 | 3.57  (2.37 – 5.38) | 2.32 x 10^-8^ |  |  |  |  |
| Kidney replaced by transplant (587) | 49 | 600 | 7.67  (3.98 – 14.78) | 2.32 x 10^-8^ |  |  |  |  |
| Nephritis; nephrosis; renal sclerosis (580) | 188 | 600 | 3.03  (2.10 – 4.37) | 5.60 x 10^-8^ |  |  |  |  |
| Shortness of breath (512.7) | 192 | 490 | 3.21  (2.18 – 4.72) | 5.76 x 10^-8^ |  |  |  |  |
| Hyperpotassemia (276.13) | 75 | 651 | 4.73  (2.83 – 7.93) | 6.03 x 10^-8^ |  |  |  |  |
| Disorders of fluid, electrolyte, and acid-base balance (276) | 243 | 651 | 2.71  (1.94 – 3.77) | 6.47 x 10^-8^ |  |  |  |  |
| Glomerulonephritis (580.1) | 90 | 600 | 4.10  (2.53 – 6.65) | 1.66 x 10^-7^ |  |  |  |  |
| Other diseases of lung (510) | 140 | 724 | 3.08  (2.08 – 4.55) | 2.73 x 10^-7^ |  |  |  |  |
| Fever of unknown origin (783) | 197 | 696 | 2.68  (1.90 – 3.78) | 3.12 x 10^-7^ |  |  |  |  |
| Acidosis (276.41) | 71 | 651 | 4.48  (2.65 – 7.58) | 3.24 x 10^-7^ |  |  |  |  |
| Anemia of chronic disease (285.2) | 87 | 557 | 4.03  (2.45 – 6.63) | 5.17 x 10^-7^ |  |  |  |  |
| Acid-base balance disorder (276.4) | 72 | 651 | 4.30  (2.55 – 7.24) | 5.68 x 10^-7^ |  |  |  |  |
| Anemia in chronic kidney disease (285.21) | 56 | 557 | 5.16  (2.85 – 9.33) | 7.38 x 10^-7^ |  |  |  |  |
| Edema (782.3) | 110 | 690 | 3.29  (2.12 – 5.11) | 1.52 x 10^-6^ |  |  |  |  |
| Other forms of chronic heart disease (414) | 30 | 790 | 9.10  (3.94 – 20.98) | 2.69 x 10^-6^ |  |  |  |  |
| Ill-defined descriptions and complications of heart disease (429) | 123 | 702 | 2.90  (1.92 – 4.37) | 4.57 x 10^-6^ |  |  |  |  |
| Other dyspnea (512.9) | 144 | 490 | 3.01  (1.96 – 4.61) | 4.74 x 10^-6^ |  |  |  |  |
| Iron deficiency anemias (280) | 90 | 557 | 3.53  (2.16 – 5.75) | 4.80 x 10^-6^ |  |  |  |  |
| Nephritis and nephropathy in diseases classified elsewhere (580.31) | 141 | 600 | 2.85  (1.90 – 4.29) | 5.01 x 10^-6^ |  |  |  |  |
| Pulmonary heart disease (415) | 64 | 785 | 3.97  (2.32 – 6.79) | 5.03 x 10^-6^ |  |  |  |  |
| Congestive heart failure; nonhypertensive (428) | 86 | 702 | 3.53  (2.15 – 5.78) | 5.63 x 10^-6^ |  |  |  |  |
| Symptoms involving cardiovascular system (429.3) | 118 | 702 | 2.88  (1.90 – 4.37) | 6.68 x 10^-6^ |  |  |  |  |
| Chronic glomerulonephritis, NOS (580.14) | 49 | 600 | 4.84  (2.57 – 9.11) | 9.97 x 10^-6^ |  |  |  |  |
| Nephritis and nephropathy without mention of glomerulonephritis (580.3) | 152 | 600 | 2.68  (1.80 – 3.99) | 1.07 x 10^-5^ |  |  |  |  |
| Disorders resulting from impaired renal function (588) | 50 | 600 | 4.61  (2.49 – 8.55) | 1.11 x 10^-5^ |  |  |  |  |
| Nephrotic syndrome without mention of glomerulonephritis (580.2) | 41 | 600 | 5.47  (2.75 – 10.89) | 1.12 x 10^-5^ |  |  |  |  |
| Carditis (420) | 88 | 823 | 3.05  (1.91 – 4.88) | 2.77 x 10^-5^ |  |  |  |  |
| Pruritis and related conditions (698) | 23 | 925 | 9.79  (3.71 – 25.84) | 3.44 x 10^-5^ |  |  |  |  |
| Iron deficiency anemias, unspecified (280.1) | 80 | 557 | 3.32  (1.99 – 5.54) | 3.83 x 10^-5^ |  |  |  |  |
| Hypovolemia (276.5) | 109 | 651 | 2.76  (1.77 – 4.30) | 6.54 x 10^-5^ |  |  |  |  |
| Nephritis and nephropathy with pathological lesion (580.32) | 50 | 600 | 4.15  (2.21 – 7.81) | 7.88 x 10^-5^ |  |  |  |  |
| Hypertensive heart disease (401.21) | 22 | 494 | 6.91  (3.27 – 22.57) | 1.01 x 10^-4^ |  |  |  |  |
| Hypopotassemia (276.14) | 78 | 651 | 3.07  (1.85 – 5.09) | 1.11 x 10^-4^ |  |  |  |  |
| Acute posthemorrhagic anemia (285.1) | 35 | 557 | 5.04  (2.42 – 10.51) | 1.22 x 10^-4^ |  |  |  |  |
| Cerebral artery occlusion, with cerebral infarction (433.21) | 28 | 841 | 5.97  (2.64 – 13.49) | 1.28 x 10^-4^ |  |  |  |  |
| Fluid overload (276.6) | 57 | 651 | 3.47  (1.96 – 6.17) | 1.52 x 10^-4^ |  |  |  |  |
| Acute-but ill-defined cerebrovascular disease (433.6) | 20 | 841 | 8.37  (3.13 – 22.43) | 1.67 x 10^-4^ |  |  |  |  |
| Cough (512.8) | 173 | 490 | 2.43  (1.61 – 3.68) | 1.71 x 10^-4^ |  |  |  |  |
| Occlusion of cerebral arteries (433.2) | 29 | 841 | 5.38  (2.43 – 11.93) | 2.30 x 10^-4^ |  |  |  |  |
| Swelling of limb (771.1) | 42 | 795 | 3.96  (2.11 – 7.44) | 2.30 x 10^-4^ |  |  |  |  |
| Respiratory failure, insufficiency, arrest (509) | 86 | 700 | 2.79  (1.71 – 4.54) | 2.50 x 10^-4^ |  |  |  |  |
| Lymphadenitis (289.4) | 56 | 706 | 3.33  (1.87 – 5.92) | 2.67 x 10^-4^ |  |  |  |  |
| Cerebrovascular disease (433) | 85 | 841 | 2.80  (1.69 – 4.65) | 4.23 x 10^-4^ |  |  |  |  |
| Superficial cellulitis and abscess (681) | 92 | 793 | 2.56  (1.61 – 4.06) | 4.30 x 10^-4^ |  |  |  |  |
| Disorders of plasma protein metabolism (270.3) | 26 | 900 | 5.52  (2.37 – 12.84) | 4.46 x 10^-4^ |  |  |  |  |
| Ascites (non malignant)  (572) | 36 | 741 | 4.26  (2.08 – 8.72) | 4.47 x 10^-4^ |  |  |  |  |
| Other venous embolism and thrombosis (452) | 89 | 741 | 2.58  (1.62 – 4.13) | 4.47 x 10^-4^ |  |  |  |  |
| Other symptoms involving abdomen and pelvis (579) | 203 | 612 | 2.04  (1.43 – 2.92) | 4.82 x 10^-4^ |  |  |  |  |
| Painful respiration (512.2) | 50 | 490 | 3.46  (1.86 – 6.44) | 5.10 x 10^-4^ |  |  |  |  |
| Hemorrhage of gastrointestinal tract (578.9) | 23 | 612 | 5.83  (2.40 – 14.12) | 5.51 x 10^-4^ |  |  |  |  |
| Heart valve disorders (395) | 68 | 794 | 2.89  (1.69 – 4.94) | 5.52 x 10^-4^ |  |  |  |  |
| Type 1 diabetes (250.1) | 25 | 783 | 5.34  (2.30 – 12.43) | 5.52 x 10^-4^ |  |  |  |  |
| Pericarditis (420.2) | 74 | 823 | 2.74  (1.65 – 4.55) | 5.52 x 10^-4^ |  |  |  |  |
| Diabetes mellitus (250) | 128 | 783 | 2.27  (1.49 – 3.45) | 6.95 x 10^-4^ |  |  |  |  |
| Disorders of calcium/phosphorus metabolism (275.5) | 38 | 885 | 3.85  (1.93 – 7.68) | 7.03 x 10^-4^ |  |  |  |  |
| Thrombocytopenia (278.3) | 89 | 590 | 2.49  (1.56 – 3.98) | 7.03 x 10^-4^ |  |  |  |  |
| Cerebral ischemia (433.3) | 38 | 841 | 3.92  (1.93 – 7.93) | 7.74 x 10^-4^ |  |  |  |  |
| Acute pulmonary heart disease (415.1) | 30 | 785 | 4.39  (2.04 – 9.46) | 8.06 x 10^-4^ |  |  |  |  |
| Respiratory failure (509.1) | 67 | 700 | 2.81  (1.64 – 4.81) | 8.06 x 10^-4^ |  |  |  |  |
| Joint effusions (741.4) | 23 | 800 | 5.46  (2.25 – 13.22) | 8.41 x 10^-4^ |  |  |  |  |
| Protein-calorie malnutrition (260) | 49 | 655 | 3.23  (1.75 – 5.97) | 8.94 x 10^-4^ |  |  |  |  |
| Pyelonephritis (590) | 29 | 538 | 4.45  (2.03 – 9.74) | 9.20 x 10^-4^ |  |  |  |  |
| Other disorders of urethra and urinary tract (597) | 203 | 633 | 1.99  (1.39 – 2.86) | 9.20 x 10^-4^ |  |  |  |  |
| Nausea and vomiting (789) | 166 | 718 | 2.00  (1.39 – 2.88) | 9.46 x 10^-4^ |  |  |  |  |
| Disorder of skin and subcutaneous tissue NOS (689) | 29 | 897 | 4.34  (1.99 – 9.49) | 1.06 x 10^-3^ |  |  |  |  |
| Abdominal pain (785) | 184 | 698 | 1.95  (1.37 – 2.79) | 1.06 x 10^-3^ |  |  |  |  |
| Disorders of protein plasma/amino-acid transport and metabolism (270) | 28 | 900 | 4.48  (2.01 – 9.95) | 1.07 x 10^-3^ |  |  |  |  |
| Purpura and other hemorrhagic conditions (287) | 95 | 590 | 2.35  (1.49 – 3.71) | 1.07 x 10^-3^ |  |  |  |  |
| Complications of cardiac/vascular device, implant, and graft (854) | 22 | 852 | 5.45  (2.19 0 13.56) | 1.18 x 10^-3^ |  |  |  |  |
| Septicemia (038) | 58 | 894 | 2.83  (1.62 – 4.95) | 1.18 x 10^-3^ |  |  |  |  |
| Bacteremia (038.3) | 58 | 894 | 2.83  (1.62 – 4.95) | 1.18 x 10^-3^ |  |  |  |  |
| Acquired foot deformities (735) | 32 | 742 | 4.02  (1.90 – 8.49) | 1.18 x 10^-3^ |  |  |  |  |
| Candidiasis (112) | 72 | 806 | 2.57  (1.54 – 4.29) | 1.23 x 10^-3^ |  |  |  |  |
| Late effects of cerebrovascular disease (433.8) | 23 | 841 | 5.43  (2.16 – 13.63) | 1.33 x 10^-3^ |  |  |  |  |
| Aplastic anemia (284) | 41 | 557 | 3.44  (1.75 – 6.75) | 1.37 x 10^-3^ |  |  |  |  |
| Cellulitis and abscess of trunk (681.7) | 22 | 793 | 5.62  (2.18 – 14.45) | 1.41 x 10^-3^ |  |  |  |  |
| Chronic kidney disease, stage III (585.33) | 57 | 600 | 2.95  (2.18 – 14.45) | 1.41 x 10^-3^ |  |  |  |  |
| Complications of transplants and reattached limbs (851) | 20 | 852 | 6.20  (2.28 – 16.87) | 1.41 x 10^-3^ |  |  |  |  |
| Inflammatory diseases of female pelvic organs (614) | 50 | 864 | 2.97  (1.63 – 5.39) | 1.45 x 10^-3^ |  |  |  |  |
| Atopic/contact dermatitis (939) | 78 | 732 | 2.47  (1.49 – 4.11) | 1.90 x 10^-3^ |  |  |  |  |
| Inflammatory disease of cervix, vagina, and vulva (614.5) | 36 | 864 | 3.43  (1.71 – 6.89) | 2.08 x 10^-3^ |  |  |  |  |
| Noninflammatory female genital disorders (619) | 55 | 867 | 2.74  (1.54 – 4.84) | 2.16 x 10^-3^ |  |  |  |  |
| Transient cerebral ischemia (433.31) | 35 | 841 | 3.62  (1.74 – 7.55) | 2.27 x 10^-3^ |  |  |  |  |
| Other pulmonary inflammation or edema (505) | 20 | 700 | 5.45  (2.02 – 13.23) | 2.32 x 10^-3^ |  |  |  |  |
| Chronic pulmonary heart disease (415.2) | 38 | 785 | 3.23  (1.64 – 6.39) | 2.78 x 10^-3^ |  |  |  |  |
| Disorders of mineral metabolism (275) | 53 | 885 | 2.71  (1.52 – 4.85) | 2.83 x 10^-3^ |  |  |  |  |
| Diseases of white blood cells (288) | 94 | 706 | 2.21  (1.39 – 3.51) | 3.02 x 10^-3^ |  |  |  |  |
| Pulmonary embolism and infarction, acute (415.11) | 28 | 785 | 3.73  (1.69 – 8.23) | 4.03 x 10^-3^ |  |  |  |  |
| Pulmonary congestion and hypostasis (503) | 26 | 700 | 3.88  (1.72 – 8.76) | 4.03 x 10^-3^ |  |  |  |  |
| Other disorders of circulatory system (459) | 31 | 794 | 3.51  (1.63 – 7.54) | 4.75 x 10^-3^ |  |  |  |  |
| Overweight, obesity, and other hyperalimentation (278) | 118 | 783 | 1.98  (1.30 – 3.00) | 4.85 x 10^-3^ |  |  |  |  |
| Chronic kidney disease, stage IV (585.34) | 29 | 600 | 3.68  (1.65 – 8.19) | 5.00 x 10^-3^ |  |  |  |  |
| Circulatory disease NEC (459.9) | 29 | 794 | 3.56  (1.61 – 7.86) | 5.87 x 10^-3^ |  |  |  |  |
| Mixed hyperlipidemia (272.13) | 75 | 737 | 2.35  (1.37 – 4.01) | 6.46 x 10^-3^ |  |  |  |  |
| Insulin pump user (250.3) | 21 | 783 | 4.25  (1.70 – 10.58) | 6.52 x 10^-3^ |  |  |  |  |
| Glaucoma (365) | 30 | 819 | 3.52  (1.57 – 7.88) | 7.53 x 10^-3^ |  |  |  |  |
| Nonrheumatic aortic valve disorders (395.2) | 23 | 794 | 4.12  (1.65 – 10.29) | 8.27 x 10^-3^ |  |  |  |  |
| Abnormal heart sounds (396) | 28 | 794 | 3.37  (1.53 – 7.43) | 8.69 x 10^-3^ |  |  |  |  |
| Vaginitis and vulvovaginitis (614.52) | 24 | 864 | 3.67  (1.57 – 8.55) | 8.78 x 10^-3^ |  |  |  |  |
| Disorders of phosphorus metabolism (275.53) | 24 | 885 | 3.96  (1.61 – 9.71) | 8.79 x 10^-3^ |  |  |  |  |
| Dermatophytosis/Dermatomycosis (110) | 37 | 806 | 2.89  (1.43 – 5.84) | 1.01 x 10^-2^ |  |  |  |  |
| Neurological disorders (292) | 75 | 793 | 2.19  (1.30 – 3.68) | 1.02 x 10^-2^ |  |  |  |  |
| Hypotension NOS (458.9) | 62 | 794 | 2.32  (1.32 – 4.06) | 1.06 x 10^-2^ |  |  |  |  |
| Symptoms involving head and neck (293) | 161 | 680 | 1.76  (1.20 – 2.57) | 1.15 x 10^-2^ |  |  |  |  |
| Hypotension (458) | 77 | 794 | 2.12  (1.27 – 3.55) | 1.30 x 10^-2^ |  |  |  |  |
| Symptoms and disorders of the joints (741) | 70 | 800 | 2.15  (1.27 – 3.63) | 1.37 x 10^-2^ |  |  |  |  |
| Disease of the oral soft tissues (528) | 27 | 902 | 3.14  (1.42 – 6.97) | 1.49 x 10^-2^ |  |  |  |  |
| Hyperlipidemia (272.1) | 148 | 737 | 1.84  (1.20 – 2.83) | 1.61 x 10^-2^ |  |  |  |  |
| Disorders of lipoid metabolism (272) | 170 | 737 | 1.73  (1.16 – 2.59) | 2.37 x 10^-2^ |  |  |  |  |
| Excessive or frequent menstruation (626.12) | 26 | 746 | 3.06  (1.33 – 7.02) | 2.55 x 10^-2^ |  |  |  |  |
| Diseases of pancreas (577) | 24 | 942 | 3.06  (1.32 – 7.10) | 2.72 x 10^-2^ |  |  |  |  |
| Chronic ulcer of skin (707) | 34 | 928 | 2.64  (1.27 – 5.51) | 2.87 x 10^-2^ |  |  |  |  |
| Diseases of esophagus (530) | 179 | 665 | 1.65  (1.13 – 2.42) | 2.94 x 10^-2^ |  |  |  |  |
| Cardiomyopathy (425) | 27 | 823 | 2.87  (1.29 – 6.41) | 2.94 x 10^-2^ |  |  |  |  |
| Acute pharyngitis (465.2) | 34 | 719 | 2.55  (1.25 – 5.23) | 3.02 x 10^-2^ |  |  |  |  |
| Disorders of function of stomach (536) | 32 | 841 | 2.60  (1.25 – 5.44) | 3.14 x 10^-2^ |  |  |  |  |
| Respiratory abnormalities (513) | 30 | 914 | 2.68  (1.25 – 5.72) | 3.18 x 10^-2^ |  |  |  |  |
| Disorders of adrenal glands (255) | 21 | 845 | 3.22  (1.30 – 7.94) | 3.21 x 10^-2^ |  |  |  |  |
| Abnormal findings on examination of urine (598) | 108 | 809 | 1.75  (1.13 – 2.71) | 3.26 x 10^-2^ |  |  |  |  |
| Gastrointestinal hemorrhage (578) | 75 | 612 | 1.94  (1.15 – 3.29) | 3.77 x 10^-2^ |  |  |  |  |
| Peripheral vascular disease (443.9) | 25 | 738 | 3.16  (1.27 – 7.90) | 3.80 x 10^-2^ |  |  |  |  |
| Encephalopathy (348.8) | 22 | 742 | 3.07  (1.26 – 7.52) | 3.81 x 10^-2^ |  |  |  |  |
| Abdominal hernia (550) | 44 | 885 | 2.26  (1.18 – 4.33) | 3.83 x 10^-2^ |  |  |  |  |
| Esophagitis, GERD, and related diseases (530.1) | 166 | 665 | 1.63  (1.10 – 2.42) | 3.86 x 10^-2^ |  |  |  |  |
| Other disease of respiratory system (519) | 23 | 906 | 2.97  (1.24 – 7.12) | 3.87 x 10^-2^ |  |  |  |  |
| Acute upper respiratory infections (465) | 110 | 719 | 1.73  (1.11 – 2.68) | 3.87 x 10^-2^ |  |  |  |  |
| Coagulation defects (286) | 146 | 590 | 1.64  (1.10 – 2.44) | 3.93 x 10^-2^ |  |  |  |  |
| Rheumatic disease of the heart valves (394) | 22 | 794 | 2.98  (1.23 – 7.21) | 4.02 x 10^-2^ |  |  |  |  |
| Non-proliferative glomerulonephritis (580.12) | 27 | 600 | 2.78  (1.21 – 6.38) | 4.13 x 10^-2^ |  |  |  |  |
| Abnormal sputum (516) | 22 | 937 | 2.97  (1.23 – 7.20) | 4.14 x 10^-2^ |  |  |  |  |
| Hemoptysis (516.1) | 22 | 937 | 2.97  (1.23 – 7.20) | 4.14 x 10^-2^ |  |  |  |  |
| Other specified nonpsychotic and/or transient mental disorders (291) | 46 | 793 | 2.22  (1.16 – 4.25) | 4.17 x 10^-2^ |  |  |  |  |
| Functional digestive disorders (564) | 91 | 705 | 1.81  (1.11 – 2.93) | 4.18 x 10^-2^ |  |  |  |  |
| Uterine leiomyoma (218.1) | 22 | 861 | 2.94  (1.22 – 7.11) | 4.18 x 10^-2^ |  |  |  |  |
| Hyposmolality and/or hyponatremia (276.12) | 37 | 651 | 2.43  (1.17 – 5.01) | 4.18 x 10^-2^ |  |  |  |  |
| Noninfectious gastroenteritis (558) | 31 | 705 | 2.50  (1.18 – 5.31) | 4.18 x 10^-2^ |  |  |  |  |
| Pancytopenia (284.1) | 30 | 557 | 2.63  (1.19 – 5.80) | 4.18 x 10^-2^ |  |  |  |  |
| Arrhythmia (427.5) | 37 | 684 | 2.39  (1.17 – 4.87) | 4.20 x 10^-2^ |  |  |  |  |
| Atherosclerosis (440) | 24 | 738 | 2.99  (1.22 – 7.37) | 4.20 x 10^-2^ |  |  |  |  |
| Chronic kidney disease, Stage I or II (585.4) | 68 | 600 | 1.96  (1.12 – 3.41) | 4.31 x 10^-2^ |  |  |  |  |
| Cardiac dysrhythmias (427) | 171 | 684 | 1.60  (1.08 – 2.36) | 4.48 x 10^-2^ |  |  |  |  |
| Dysphagia (532) | 66 | 665 | 1.92  (1.11 – 3.32) | 4.75 x 10^-2^ |  |  |  |  |
| Known or suspected fetal abnormality affecting management of mother (655) | 61 | 897 | 0.46  (0.24 – 0.89) | 4.81 x 10^-2^ |  |  |  |  |
| Sepsis and SIRS (994) | 66 | 888 | 1.88  (1.10 – 3.20) | 4.82 x 10^-2^ |  |  |  |  |
| Symptoms involving respiratory system and other chest symptoms (519.9) | 20 | 906 | 2.99  (1.18 – 7.56) | 4.88 x 10^-2^ |  |  |  |  |
| Immunity deficiency (279.1) | 24 | 567 | 2.76  (1.17 – 6.51) | 4.92 x 10^-2^ |  |  |  |  |
| Benign neoplasm of uterus (218) | 23 | 861 | 2.73  (1.15 – 6.49) | 4.92 x 10-2 |  |  |  |  |

^£^Phenotype present indicates subjects who had the code listed on at least 2 instances vs. phenotype absent indicates subjects who did not have the code or related codes. Subjects with 1 instance of a code are excluded, so the total number of subjects for each PheWAS code does not add up to the 1097 SLE subjects.

*Codes listed met the false discovery rate of 0.05.

**
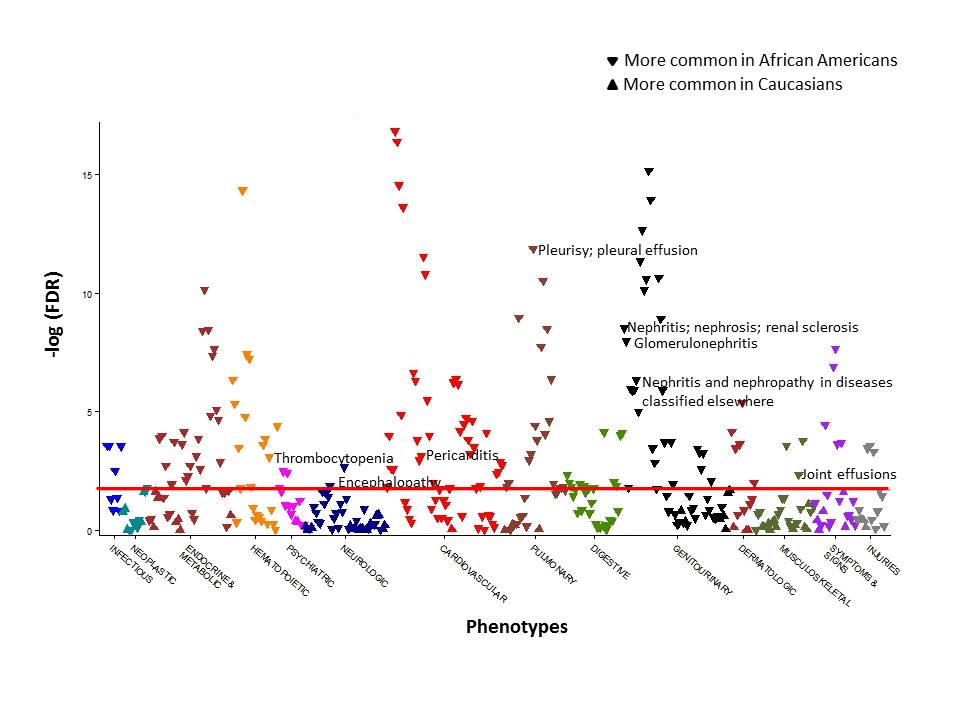
**

**Figure S1. Selected SLE disease criteria codes in the PheWAS of African Americans and Cauasians with SLE.**

The x axis represents the PheWAS codes that are mapped to ICD-9 codes, organized and color-coded by organ system. The y axis represents the level of significance. Each triangle represents a PheWAS code. African Americans are the reference group. Triangles pointing down represent codes more common in African Americans. Triangles pointing up represent codes more common in Caucasians. The PheWAS was adjusted for age and sex, and the horizontal red line represents the false discovery rate (FDR) of 0.05. There were 163 codes that met the FDR of 0.05 with the most significant codes related to SLE disease criteria labeled.

**Table S2. Selected SLE criteria codes from the PheWAS of African Americans and Caucasians with SLE.**

| **PheWAS Code Name**  (Code Number) | **Phenotype Present**  (≥ 2 instances of the code)^£^ | **Phenotype**  **Absent**  (0 instances of the code)^£^ | **Adjusted Odds Ratio for current age and sex**  **(95% CI)** | **False Discovery Rate p*** |
| --- | --- | --- | --- | --- |
| Pleurisy/pleural effusion (507) | 127 | 700 | **African American:** 4.39 (2.92 – 6.62)  Caucasian: 1.00 (ref) | 5.53 x 10^-11^ |
| Nephritis; nephrosis; renal sclerosis  (512.7) | 188 | 600 | 3.03 (2.10 – 4.37) | 5.60 x 10^-8^ |
| Glomerulonephritis (580.1) | 90 | 600 | 4.10  (2.53 – 6.65) | 1.66 x 10^-7^ |
| Nephritis and nephropathy in diseases classified elsewhere (580.31) | 141 | 600 | 2.85  (1.90 – 4.29) | 5.01 x 10^-6^ |
| Pericarditis (420.2) | 74 | 823 | 2.74  (1.65 – 4.55) | 5.52 x 10^-4^ |
| Thrombocytopenia (287.3) | 89 | 590 | 2.49  (1.56 – 3.98) | 7.03 x 10^-4^ |
| Joint effusions (741.4) | 23 | 800 | 5.46  (2.25 – 13.22) | 8.41 x 10^-4^ |
| Aplastic anemia (284) | 41 | 557 | 3.44  (1.75 – 6.75) | 1.37 x 10^-3^ |
| Symptoms and disorders of the joints (741) | 70 | 800 | 2.15  (1.27 – 3.63) | 0.01 |
| Encephalopathy, not elsewhere classified (348.8) | 22 | 742 | 3.07  (1.26 – 7.52) | 0.04 |
| Pancytopenia (284.1) | 30 | 557 | 2.63  (1.19 – 5.80) | 0.04 |

^£^Phenotype present indicates subjects who had the code listed on at least 2 instances vs. phenotype absent indicates subjects who did not have the code or related codes. Subjects with 1 instance of a code are excluded, so the total number of subjects for each PheWAS code does not add up to the 1097 SLE subjects.

*Codes listed met the false discovery rate of 0.05.

**Table S3. Selected codes related to renal, cardiovascular disease, and infection from the PheWAS of African American SLE cases compared to matched African American controls.**

| **PheWAS Code Name**  (Code Number) | **Phenotype Present**  (≥ 2 instances of code)^£^ | **Phenotype**  **Absent**  (0 instances of code)^£^ | **Odds Ratio**  **(95% CI)** | **False Discovery Rate p*** |
| --- | --- | --- | --- | --- |
| **Renal codes** |  |  |  |  |
| Renal failure (585) | 227 | 1315 | **African American SLE case:** 9.55  (6.91 – 13.18)  **African American control**: 1.00 (ref) | 2.26 x 10^-40^ |
| Chronic renal failure [CKD] (585.3) | 162 | 1315 | 9.36  (6.52 – 13.45) | 1.36 x 10^-31^ |
| Renal dialysis (585.31) | 76 | 1315 | 25.37  (14.92 – 43.15) | 6.26 x 10^-31^ |
| End stage renal disease (585.32) | 89 | 1315 | 14.53  (9.15 – 23.07) | 5.13 x 10^-28^ |
| Kidney replaced by a transplant (587) | 50 | 1315 | 20.06  (10.84 – 37.13) | 2.78 x 10^-20^ |
| **Cardiovascular codes** |  |  |  |  |
| Hypertensive heart and/or renal disease (401.2) | 135 | 904 | 8.08  (5.39 – 12.11) | 1.78 x 10^-22^ |
| Hypertension (401) | 646 | 904 | 3.26  (2.46 – 4.33) | 4.24 x 10^-15^ |
| Other venous embolism and thrombosis (452) | 85 | 1382 | 5.72  (3.63 – 8.99) | 4.74 x 10^-13^ |
| Congestive heart failure (428) | 99 | 1304 | 4.36  (2.81 – 6.77) | 4.17 x 10^-10^ |
| Other forms of chronic heart disease (414) | 41 | 1468 | 6.54  (3.48 – 12.29) | 3.06 x 10^-8^ |
| Peripheral vascular disease (443) | 39 | 1515 | 6.43  (3.37 – 12.27) | 8.84 x 10^-8^ |
| Pulmonary embolism and infarction, acute (415.11) | 33 | 1453 | 6.21  (3.07 – 12.54) | 1.73 x 10^-6^ |
| Cerebrovascular disease (433) | 97 | 1528 | 3.01  (1.92 – 4.70) | 6.08 x 10^-6^ |
| Atrial fibrillation and flutter (427.2) | 40 | 1318 | 3.89  (2.01 – 7.53) | 1.91 x 10^-4^ |
| Cardiomyopathy (425) | 46 | 1550 | 2.75  (1.42 – 5.32) | 6.83 x 10^-3^ |
| Atherosclerosis (440) | 29 | 1515 | 3.05  (1.37 – 6.79) | 0.02 |
| **Infection codes** |  |  |  |  |
| Pneumonia (480) | 139 | 1315 | 5.77  (3.97 – 8.39) | 8.66 x 10^-19^ |
| Bacteremia (038.3) | 46 | 1604 | 8.45  (4.62 – 15.44) | 3.46 x 10^-11^ |
| Sepsis and SIRS (994) | 60 | 1591 | 5.13  (3.03 – 8.67) | 7.49 x 10^-9^ |
| Candidiasis (912) | 79 | 1379 | 3.88  (2.41 – 6.24) | 1.34 x 10^-7^ |
| Pyelonephritis (590) | 37 | 1170 | 6.99  (3.52 – 13.88) | 1.52 x 10^-7^ |
| Cellulitis and abscess of trunk (681.7) | 40 | 1341 | 3.55  (1.84 – 6.85) | 5.41 x 10^-4^ |
| Osteomyelitis, periostitis, and other infections involving bone (710) | 23 | 1495 | 4.29  (1.83 – 10.04) | 2.29 x 10^-3^ |
| Superficial cellulitis and abscess (681) | 158 | 1341 | 1.94  (1.31 – 2.87) | 2.66 x 10^-3^ |

^£^Phenotype present indicates subjects who had the code listed on at least 2 instances vs. phenotype absent indicates subjects who did not have the code or related codes. Subjects with1 instance of a code are excluded, so the total number of subjects for each PheWAS code does not add up to the 1695 total of SLE subjects and matched controls.

*Codes listed met the false discovery rate of 0.05.

**Table S4. Selected codes related to SLE criteria from the PheWAS of African American SLE cases and matched African American controls.**

| **PheWAS Code Name**  (Code Number) | **Phenotype Present**  (≥ 2 instances of the code)^£^ | **Phenotype**  **Absent**  (0 instances of the code)^£^ | **Odds Ratio for current age and sex**  **(95% CI)** | **False Discovery Rate p*** |
| --- | --- | --- | --- | --- |
| Nephritis; nephrosis; renal sclerosis (580) | 106 | 1315 | **African American SLE case:** 50.53  (29.40 – 86.86)  **African American control**: 1.00 (ref) | 4.77x 10^-43^ |
| Pleurisy; pleural effusion (507) | 115 | 1389 | 12.41  (8.24 – 18.70) | 1.83 x 10^-31^ |
| Glomerulonephritis (580.1) | 56 | 1315 | 72.36  (32.04 – 163.39) | 2.88 x 10^-23^ |
| Thrombocytopenia (287.3) | 73 | 1400 | 11.91  (7.26 – 19.54) | 2.89 x 10^-21^ |
| Pericarditis (420.2) | 49 | 1550 | 24.15  (12.14 – 48.01) | 2.09 x 10^-18^ |
| Aplastic anemia (284) | 36 | 961 | 9.62  (4.84 – 19.11) | 7.39 x 10^-10^ |
| Encephalopathy (348.8) | 22 | 1396 | 7.44  (3.17 – 17.44) | 1.67 x 10^-5^ |
| Joint effusions (741.4) | 39 | 1441 | 3.30  (1.69 – 6.45) | 1.47 x 10^-3^ |

^£^Phenotype present indicates subjects who had the code listed on at least 2 instances vs. phenotype absent indicates subjects who did not have the code or related codes. Subjects with1 instance of a code are excluded so the total number of subjects for each PheWAS code does not add up to the 1695 total of SLE subjects and matched controls.

*Codes listed met the false discovery rate of 0.05.

**Table S5. Selected codes from the PheWAS of Caucasian SLE cases compared to matched Caucasian controls.**

| **PheWAS Code Name**  (Code Number) | **Phenotype Present**  (≥ 2 instances of code)^£^ | **Phenotype**  **Absent**  (0 instances of code)^£^ | **Odds Ratio**  **(95% CI)** | **False Discovery Rate p*** |
| --- | --- | --- | --- | --- |
| **Renal codes** |  |  |  |  |
| Renal failure (585) | 296 | 3588 | **Caucasian SLE case:** 6.16  (4.80 – 7.91)  **Caucasian control**: 1.00 (ref) | 7.91 x 10^-44^ |
| Chronic renal failure [CKD] (585.3) | 173 | 3588 | 7.88  (5.74 – 10.82) | 3.73 x 10^-35^ |
| Nephritis; nephrosis; renal sclerosis (580) | 106 | 3588 | 128.06  (55.43 – 295.84) | 4.88 x 10^-28^ |
| Acute renal failure (585.1) | 151 | 3588 | 5.81  (4.15 – 8.13) | 7.24 x 10^-23^ |
| Renal dialysis  (585.31) | 35 | 3588 | 8.32  (4.22 – 16.41) | 1.08 x 10^-8^ |
| **SLE-related codes** |  |  |  |  |
| Myalgia and myositis unspecified (770) | 349 | 3645 | 8.30  (6.57 – 10.49) | 2.90 x 10^-67^ |
| Other immunological findings (279.7) | 148 | 3757 | 51.14  (31.78 – 82.31) | 1.65 x 10^-56^ |
| Erythematous conditions (695) | 161 | 3648 | 14.01  (9.91 – 19.81) | 5.01 x 10^-48^ |
| Coagulation defects (286) | 175 | 3568 | 7.38  (5.39 – 10.11) | 1.60 x 10^-33^ |
| Malaise and fatigue (798) | 934 | 2672 | 3.09  (2.57 – 3.71) | 8.56 x 10^-32^ |
| Pain in joint (745) | 1062 | 2635 | 3.10  (2.58 – 3.73) | 8.56 x 10^-32^ |
| Thrombocytopenia (287.3) | 106 | 3568 | 5.41  (3.63 – 8.08) | 3.52 x 10^-15^ |
| Pericarditis (420.2) | 52 | 4026 | 11.10  (6.17 – 19.96) | 1.88 x 10^-14^ |
| Pleurisy; pleural effusion (507) | 183 | 3678 | 2.48  (1.78 – 3.45) | 5.29 x 10^-7^ |

^£^Phenotype present indicates subjects who had the code listed on at least 2 instances vs. phenotype absent indicates subjects who did not have the code or related codes. Subjects with1 instance of a code are excluded, so the total number of subjects for each PheWAS code does not add up to the 1695 total of SLE subjects and matched controls.

*Codes listed met the false discovery rate of 0.05.

**Table S6. Conditional logistic regression models with SLE cases and matched controls.**

| **PheWAS code** | **Odds ratio (OR) for SLE disease status***  **95% Confidence Interval**  **p value** |
| --- | --- |
| **Cardiovascular codes** |  |
| Hypertension | 2.18  (1.86 – 2.55)  p < 0.01 |
| Congestive heart failure | 2.33  (1.73 – 3.13)  p = 2.2 x 10^-8^ |
| Cerebrovascular disease | 1.76  (1.34 – 2.32)  p = 5.8 x 10^-5^ |
| Cardiac dysrhythmias | 1.63  (1.34 – 1.97)  p = 9.0 x 10^-7^ |
| **Renal codes** |  |
| Chronic kidney disease | 7.04  (5.73 – 8.65)  p < 0.0001 |
| End stage renal disease | 8.00  (5.43 – 11.80)  p < 0.001 |
| Renal transplant | 7.61  (4.70-12.33)  p = 1.1 x 10^-16^ |
| **Infectious codes** |  |
| Pneumonia | 3.86  (2.98 – 5.01)  p < 0.00001 |
| Bacteremia/sepsis | 6.10  (4.05 – 9.19)  p < 0.001 |

*Odds ratio for conditional logistic regression model with PheWAS code as the outcome and covariates including SLE, age, sex, and race.
